# Solution-Phase vs Surface-Phase Aptamer-Protein Affinity from a Label-Free Kinetic Biosensor

**DOI:** 10.1371/journal.pone.0075419

**Published:** 2013-09-17

**Authors:** Camille Daniel, Yoann Roupioz, Didier Gasparutto, Thierry Livache, Arnaud Buhot

**Affiliations:** 1 Laboratoire Structure et Propriétés d’Architectures Moléculaires, UMR 5819 CEA/CNRS/UJF, Institut Nanosciences et Cryogénie, Grenoble, France; 2 Service de Chimie Inorganique et Biologique, UMR-E3 CEA/UJF, Institut Nanosciences et Cryogénie, Grenoble, France; Northeastern University, United States of America

## Abstract

Aptamers are selected DNA ligands that target biomolecules such as proteins. In recent years, they are showing an increasing interest as potential therapeutic agents or recognition elements in biosensor applications. In both cases, the need for characterizing the mating between the target and the aptamer either in solution or immobilized on a surface, is pressing. In this context, we have developed a kinetic biosensor made of micro-arrayed anti-thrombin aptamers to assess the kinetic parameters of this interaction. The binding of label-free thrombin on the biosensor was monitored in real-time by Surface Plasmon Resonance imaging. Remarkable performances were obtained for the quantification of thrombin without amplification (sub-nanomolar limit of detection and linear range of quantification to two orders of magnitude). The independent determinations of both the solution- and surface-phase affinities, respectively K_D_^Sol^ and K_D_^Surf^, revealed distinct values illustrating the importance of probes, targets or surface interactions in biosensors. Interestingly, K_D_^Surf^ values depend on the aptamer grafting density and linearly extrapolate towards K_D_^Sol^ for highly diluted probes. This suggests a lesser impact of the surface compared to the probe or target cooperativity interactions since the latter decrease with a reduced grafting density.

## Introduction

Aptamers are short oligonucleotides selected *in vitro* for their ability to bind with high affinity and specificity [1] to a wide range of target ligands especially proteins [2], [3]. Due to their high stability, specificity and low cost, they are gaining interest as an ideal recognition element in biosensor design. They have been employed in a large variety of sensing technologies [4], [5], [6] and have shown a potential for therapeutic applications [7]. In order to optimize aptamer-based technologies, there is a pressing need to characterize the interaction between the target and the aptamer either in solution or bound to the surface of a biosensor.

Different methods exist for the determination of affinities or dissociation constants (K_D_) for aptamer-protein complexes: Enzyme-linked Aptamer Assays (ELAA) [8], chromatography [9], capillary electrophoresis [10], [11], NMR [12], [13], colorimetry [14], fluorescence anisotropy [15], [16] or ionic current measurement through aptamer-modified biological nanopores [17] to mention a few. However, they often lead to a great variability of K_D_ values due to the parameters of the technique [18], as the eventual need for the labeling or the anchoring to a surface of one partner.

In the specific case of biosensors where probes are immobilized on a surface, the calculated affinities may suffer from the heterogeneity due to the grafting [19], [20], [21], probe accessibility [22] or the distance between multi-valent probes [23]. For example, in the case of DNA microarrays, more than 10 orders of magnitude differences were observed between solution-phase and surface-phase affinities for the hybridization of complementary strands [24]. For ligand library screening, the ranking of ligand affinities issued from protein microarrays may even differ from solution-phase values [25].The large variability of K_D_ for aptamer-protein complexes observed between techniques may suggest that the grafting on a surface (sensors, chips, membranes, beads…) of one partner influences the affinity [26] even though no precise and quantitative studies have characterized the effect of grafting density in detail.

Here we describe a general, simple and rapid approach to monitor the protein binding on an aptamer-based biosensor and independently assess both the aptamer-protein solution- and surface-phase affinities. The main advantages of our approach are the facts that the determination of the solution-phase affinity (i) is done before reaching the equilibrium state on the biosensor, which is generally time consuming especially at low concentrations of proteins and (ii) is independent of a surface-phase model of adsorption. In the later case, the Langmuir model is usually considered for simplicity, but the use of more sophisticated models may be required to take into account mutual interactions, heterogeneity and loss of accessibility of the probes [27].

**Figure 1:**
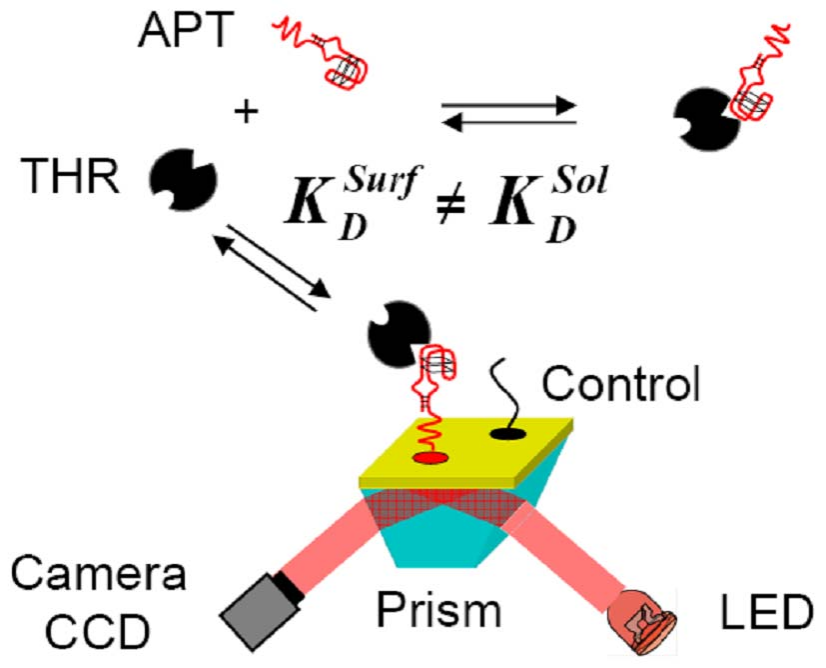
General scheme of the aptamer kinetic biosensor coupled to SPRi detection. The surface-phase dissociation constant K_D_^Surf^ of the interaction between thrombin (THR) and grafted aptamer (APT) differs from K_D_^Sol^, the solution-phase value, determined from competition assays.

A proof-of-principle label-free kinetic biosensor was developed, using the two advantageous traits of Surface Plasmon Resonance imaging (SPRi) at their best [28], [29], [30]: the label-free and real-time detection of molecular interactions occurring on the biosensor surface. State-of-the-art performances for a label-free technique without amplification were obtained: a sub-nanomolar limit of detection (LOD  =  100 pM) with a linear range of quantification (LROQ) of two orders of magnitude while a rapid detection (less than 10 min) and a strong selectivity were maintained (very low level of non-specific adsorption in presence of a large excess of competitive proteins). The independent determinations of K_D_^Sol^ and K_D_^Surf^, respectively the solution- and surface-phase affinities, revealed distinct values illustrating the importance of probes, targets or surface interactions in biosensors (Figure 1).

For this purpose, an aptamer (hereafter called APT) selected against thrombin protein [31] was considered as the recognition element. Thrombin is a human protein which plays a major role in the blood coagulation cascade by transforming the soluble protein fibrinogen to insoluble filaments of fibrin forming the fibrin gel. Moreover, it intervenes at different times in the cascade and can interact with many partners and substrates [32] justifying the development of aptamers against thrombin for therapeutic applications as well as for quantification purposes in biosensors. For these reasons, thrombin was the first protein chosen for DNA aptamer selection two decades ago [33]. Five years later another 29-oligonucleotide sequence (APT  =  5′-AGT-CCG-TGG-TAG-GGG-AGG-TTG-GGG-TGA-CT-3′), capable of inhibiting thrombin-catalyzed fibrin clot formation *in vitro*, has been reported to bind to thrombin with higher affinity [31]. However, the values of the K_D_ reported by several groups are varying by several orders of magnitude depending on the experimental techniques considered: from 0.5 nM, determined by the team who has selected the aptamer using nitrocellulose filter retention; to about 100 nM by surface plasmon resonance analysis [34] and up to 255 nM based on capillary electrophoresis studies [35].

## Materials and Methods

### Reagents and chemicals

Human alpha-thrombin (THR) was purchased from Haematologic Technologies Inc. (Essex Junction, VT, USA). Bovine serum albumin (BSA), cytochrome c (cyt c), 11-mercapto-undecanoic acid, *N*,*N*′-dicyclohexylcarbodiimide (DCC), *N*-hydroxysuccinimide (NHS), dimethylformamide (DMF) and all the reagents for buffers were purchased from Sigma-Aldrich (France). CH_3_O-PEG-SH (MW 2000 Dalton) was purchased from Rapp Polymere GmbH (Germany). All chemicals were used without further purification. The buffer used for DNA aptamers spotting was 1 M HK_2_PO_4_ solution, pH 9.25, the buffer used in the assays (running buffer), was 20 mM Tris-HCl, 1 mM MgCl_2_, 120 mM NaCl, and 10 mM KCl, pH 7.4.

All the oligonucleotides (APT: 5′-C_6_-(T_10_)-AGT-CCG-TGG-TAG-GGG-AGG-TTG-GGG-TGA-CT-3′ and Control: 5′-C_6_-(T_10_)-GAC-CAT-CGT-GCG-GGT-AGG-TAG-ACC) were purchased from Eurogentec (France) with a primary amine modification at the 5’-position (5’ Amino Modifier C6) and a 10-thymine spacer before the sequence of interest.

### Micro-array fabrication

Our kinetic biosensor is an aptamer microarray composed of different spots containing either the aptamer APT, a control sequence or mixtures of both (Figure 1). Oligonucleotides are grafted through formation of a thiol self-assembling monolayer (SAM) on gold-coated glass prisms (Horiba Scientific-GenOptics, France). We reproduced a previously described grafting protocol [36] using a piezoelectric dispensing system to depose droplets of solutions containing micromolar concentrations of oligonucleotide and polyethylene-glycol (PEG) both modified by thiols.

First, a thiol functionality was introduced onto the DNA oligonucleotides by conjugation with an activated NHS ester following a protocol previously described [37]. Probe oligonucleotides (2 nmoles) containing a primary amine group at their 5′-end were conjugated to HS-C_11_-NHS (160 nmoles) in PBS pH 8.0 for 1 hour at room temperature. After purification by size exclusion chromatography (Illustra NAP-5 Columns kit, GE Healthcare) the purified HS-oligonucleotides were re-suspended in HK_2_PO_4_ buffer and the DNA concentration was assessed by absorbance measurement at 260 nm.

Before thiol SAM formation, glass prisms recovered by a gold surface were cleaned by plasma treatment (0.6 mbar, 75% Oxygen, 25% Argon, power 40 W, 6 min) in a plasma generator (Femto, Diener Electronic, Germany). The HS-oligonucleotides were arrayed on the gold surface by droplet deposition using a piezoelectric dispensing system (Siliflow, France): droplets of approximately 4 nL of 10 or 20 µM thiol-modified DNA probes and 10 µM of methoxy-functionalised thiolated PEG (CH_3_O-PEG-SH) were deposited under a controlled atmosphere of 85% humidity. They were left on in the humid atmosphere at room temperature for 30 min to allow for a thiol mono-layer to auto-assemble on the surface. After overnight drying on the bench, prisms were thoroughly rinsed with deionised water and dried under an argon stream for few seconds.

The PEG molecules reduce the non-specific binding of proteins and help to space the aptamers on the surface to increase accessibility. After the self-assembled monolayer formation, the probe density ranges from σ  =  5.3±0.4 to 7.9±0.7 pmol/cm^2^ depending on the initial concentration of thiol-aptamer in the deposited solution (either 10 or 20 µM). After aptamer grafting, the biosensor is rinsed and loaded into the SPR imaging device where a fluidic system allows for the control of both the running buffer flow rate and target solution injections.

### Assessment of the probe grafting density

The modification of the sequence which served for the assessment of grafting density (5′-C_6_-(T_10_)-GGT-TGG-TGT-GGT-TGG-3’) was made on the thymine nucleobase instead of the phosphate group (5’ Amino Modifier C6 dT), aiming to further label the sequence with P32. Considering that the distance between the modification of the oligonucleotide and the thiol group responsible of the grafting on the gold surface remains the same in both cases, this difference is not expected to modify significantly the grafting density.

HS-oligonucleotides (100 pmol) were labeled at the 5'-terminus with 10 µCi [γ-^32^P]ATP (2 pmol, 10 mCi/mL) Perkin-Elmer (Courtaboeuf, France) upon incubation with T_4_ polynucleotide kinase (10 units, New England Biolabs) in 30 µL of supplied buffer at 37 °C for 30 min. The reaction was quenched by addition of 1 µL of a 0.5 M EDTA solution (pH 8). Unincorporated [γ-^32^P]-ATP was removed by purification of the oligonucleotide on a MicroSpin column (Pharmacia, Uppsala, Sweden).

Labeled oligonucleotides were then grafted on gold coated glass slides (7×11 mm^2^). 10 µL of spotting solution (HK_2_PO_4_ buffer containing a mixture of 16 pmol ^32^P-labeled and non-labelled oligonucleotides at various final concentrations from 0.1 to 30 µM and 10 µM of methoxy-functionalised thiolated PEG) was spread on the slides and covered with glass coverslips. Measurements of radioactivity were realized with a radioactive counter (MIP-10 ictometer from Nardeux; Loches, France) and slides were left overnight in a humid atmosphere to prevent drying. The next day they were rinsed with deionised water before radioactivity was measured again. The values of probe grafting density were measured from triplicates for each concentration of aptamers used in the grafting solution (Table 1).

**Table 1:** Values of aptamer grafting density determined from various concentration of aptamers in grafting solution (c  =  0.1, 1, 10, 20 and 30 µM).

	Aptamer grafting density (pmol/cm^2^)
c = 0.1 µM	1.3±0.1
c = 1 µM	3.2±0.7
c = 10 µM	5.3±0.4
c = 20 µM	7.9±0.7
c = 30 µM	8.3±1.6

The data represents the mean ± standard deviation (S.D.) obtained from triplicates.

### Micro-arrays with controlled aptamer grafting densities

The determination of the absolute aptamer grafting density from radioactive quantification confirmed its non-linear variation with an increasing concentration of aptamers in the grafting solution and a saturation value around 8 pmol/cm^2^ for concentrations above 20 µM. However, for the determination of the surface-phase affinity dependence on grafting density, a well controlled aptamer density was needed. In order to obtain such microarrays with controlled grafting density spots, we mixed the aptamers and the control sequences in various proportions at a constant total concentration of 20 μM before droplet deposition. Different ratios of aptamers (100%, 85%, 75%, 50%, 25%) were used leading to the following aptamer grafting densities: 8, 6.8, 6, 4, 2 pmol/cm^2^ respectively. In order to verify the conservation of the different ratios at the surface of the biochip after grafting, hybridization signals following the injection of the complementary sequence to APT were compared between the different spots. The proportionality between the signal and the aptamer concentration in spotting solution proved the success of the method (data not shown).

### Thrombin detection

Thrombin (alone or in combination with the aptamer) was co-injected with a large excess of cyt c (500 nM). For the calibration curve of the kinetic biosensor, a series of injections at different concentrations of thrombin (from 0.2 to 50 nM) diluted in the running buffer were performed on the microarray. The injections were stopped after twelve minutes. Then, several co-injections of 50 nM THR and varying concentrations (38 – 45 – 76 – 90 or 190 nM) of aptamer were carried out with the same flowing parameters. The free thrombin in solution was determined from the calibration curve previously obtained. For the surface-phase affinity determination, longer injections of various concentrations of thrombin were conducted (1.25, 2.5, 5, 10, 20, 50 nM) up to more than one hour for the lowest concentration in order to reach the equilibrium level. The surface was regenerated before each thrombin injection by flushing a 1 M NaCl solution for 8 min on the microarray.

## Results and Discussion

### Kinetic biosensor performances

**Figure 2:**
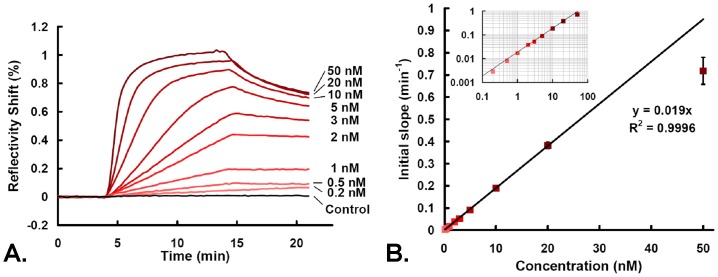
Performance of the aptamer kinetic biosensor for thrombin detection. A. Sensorgrams obtained on the kinetic biosensor for various injections of thrombin (concentrations between 200 pM and 50 nM from bottom to top). Each curve represents the averaged signal (Reflectivity shift *ΔR*) of 3 replicates of APT spots with subtraction of a control spot as function of time. B. Calibration curve of the initial slopes obtained from the first few minutes of the association phase in the sensorgrams as function of the thrombin concentration. The linear fit extends over two-order of magnitude. Inset: Same data plotted in a log-log scale.

The interaction between the thrombin and the aptamer on the microarray was monitored in real-time during injections of thrombin at different concentrations (Figure 2A). To assess the signal specificity, the thrombin was co-injected with a large excess of cytochrome c (500 nM). The equilibrium state is obtained when the signal is stabilized. As observed on Figure 2A, the equilibrium time may last from a few minutes up to hours for the lowest concentrations. Interestingly, the exploitation of the short time interaction kinetics to determine the protein concentration revealed far superior performances than the equilibrium signal handling [38], [39]. In fact, a precise calibration curve may be obtained by plotting the initial slope of the signal at the beginning of the injection against the concentration (the first few seconds should be disregarded to ensure a complete reshuffling of the solution above the biosensor). Such calibration curve provides a wide LROQ from 0.2 to 20 nM and a sub-nanomolar LOD (3 times the standard deviation of the control signal) of 100 pM (Figure 2B). The relative error on the concentration Δc/c is reduced (lower than 5%) on the whole LROQ allowing a precise quantification of the thrombin protein. Moreover, the time of analysis for THR detection is considerably reduced since only the first few minutes of the injection are necessary for the determination of the initial slope and consequently the determination of protein concentration in solution.

### Solution-phase affinity

**Figure 3:**
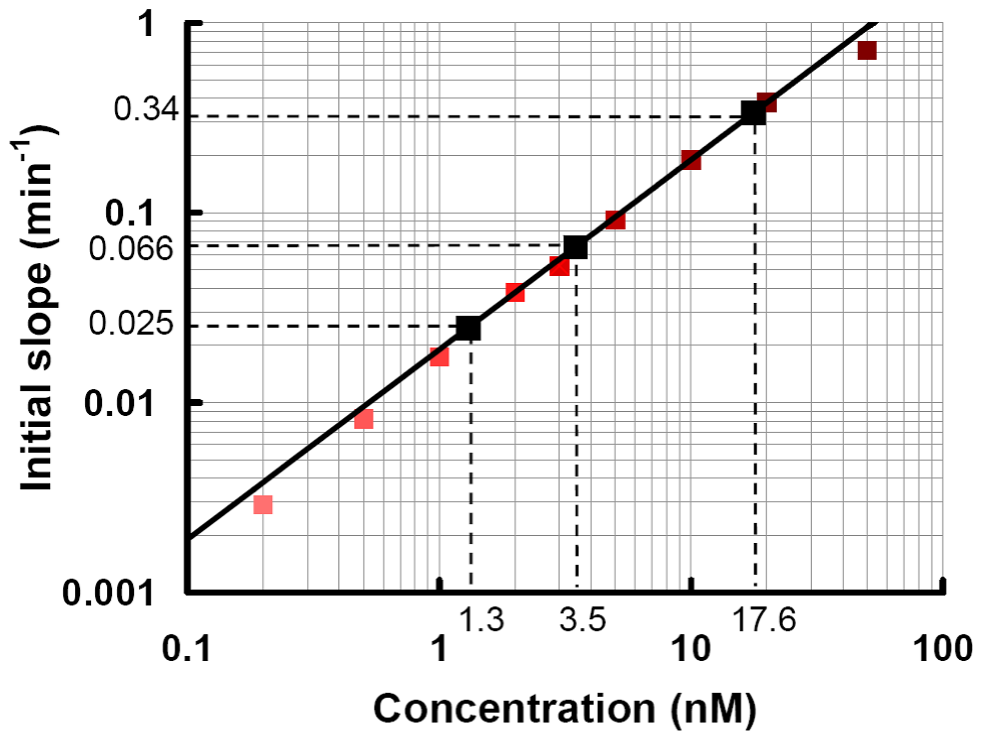
Initial slopes of signal monitored during competition assays (black dots) reported on the calibration curve (Figure 2B). The concentration of free (unbound) thrombin is determined from which the solution-phase affinity K_D_^Sol^ is deduced.



This kinetic biosensor was used to quantify the concentration of free thrombin in competition assays (Figure 3). Thrombin was incubated at a fixed concentration (c_THR_  =  50 nM) with varying concentrations (c_APT_  =  38, 45, 76, 90 and 190 nM) of aptamer in solution. From the measured concentrations [THR] of free (unbound) thrombin (Figure 3), we calculated the solution-phase dissociation constant between thrombin and aptamer:

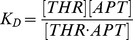



with [THR APT]  =  c_THR_ – [THR], the equilibrium concentration of thrombin bound to aptamers in solution and [APT]  =  c_APT_ – [THR APT], the concentration of free aptamers in solution.

The K_D_^Sol^ values determined from various total concentrations of aptamers (c_APT_) and different aptamer grafting densities (σ) (Table 2) are all consistent with each other. As expected, the values obtained from the two aptamer grafting densities were not statistically different (p-value  =  0.37). Furthermore, the averaged value K_D_^Sol^  =  3.16±1.16 nM is in strong agreement with the K_D_  =  3.84±0.68 nM recently published in the literature using back-scattering interferometry, a label-free technique in solution [40]. Thus this confirms the ability to access the solution-phase affinity of protein-aptamer with our method, independently of the aptamer grafted density obtained on the biosensor (as long as reasonable performances for protein detection are maintained).

**Table 2:** Values of K_D_^Sol^ determined from various total concentration of co-injected aptamers (c_APT_  =  38, 45, 76, 90 and 190 nM) and different aptamer grafting density (σ = 5.3 and 7.9 pmol cm^-2^).

	K_D_^Sol^ (nM)	
	σ = 5.3 pmol cm^-2^	σ = 7.9 pmol cm^-2^
c_APT_ = 38 nM	3.08±0.62	3.83±0.40
c_APT_ = 45 nM	4.94±0.22	4.70±1.27
c_APT_ = 76 nM	2.19±0.04	1.67±0.07
c_APT_ = 90 nM	2.05±0.20	2.46±0.29
c_APT_ = 190 nM	2.88±0.12	3.77±0.09

The data represents the mean ± S.D. obtained from triplicates. Values given for respectively cAPT  =  38, 76 and 190 nM and cAPT  =  45 and 90 nM were obtained from two different kinetic biosensors.

### Surface-phase affinity

**Figure 4:**
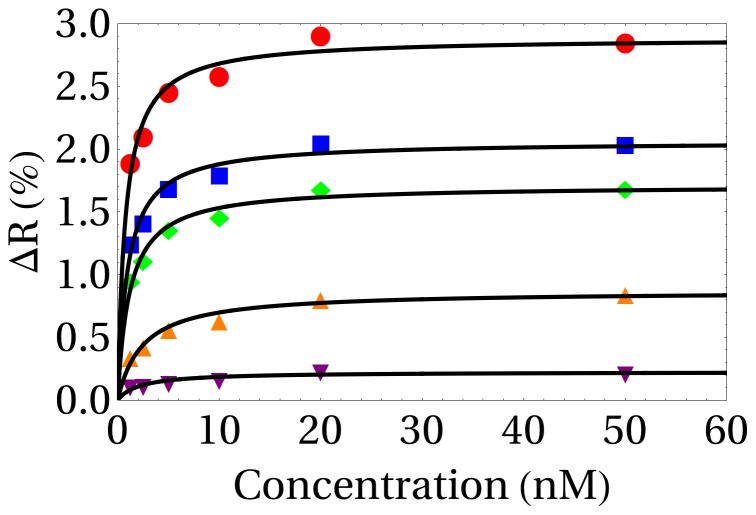
Thrombin interactions measured at equilibrium from SPRi on aptamer spots with various grafting densities as function of thrombin concentration. From bottom to top, the grafting densities are respectively 2, 4, 6, 6.8 and 8/cm^2^. Langmuir fits for the extrapolation of the surface-phase affinities are represented by black curves.

Measurement of dissociation binding constants (K_D_^Surf^) of probes to targets on surfaces (biosensors, membranes or beads) may differ from the solution-phase affinity K_D_^Sol^ due to interactions with the surface or interactions between the probes or the targets because of the proximity of the probes on the surface. The surface interactions are expected to be independent on the probe grafting density, in contrary to the interactions between probes or targets due to the relative proximity of the probes on the surface. Generally, K_D_^Surf^ is determined by fitting the Langmuir adsorption isotherm on equilibrium signals obtained following long injection times (Figure 4). Each dot on Figure 4 corresponds to the SPR signal obtained at equilibrium of several injections of thrombin on aptamer spots of varying density (2, 4, 6, 6.8, 8 pmol/cm^2^ from bottom to top) corrected by the signal obtained on control spots. Thrombin was delivered in a back and forth mode (15 µL dispensed volume, 10 µL aspirated volume, 50 µL/min flow rate) to access sufficiently long injection times (up to more than one hour for the lowest concentration). The black curves represent the non-linear Langmuir fit from which the K_D_^Surf^ are determined.

### Surface-phase vs Solution-phase affinities

**Figure 5:**
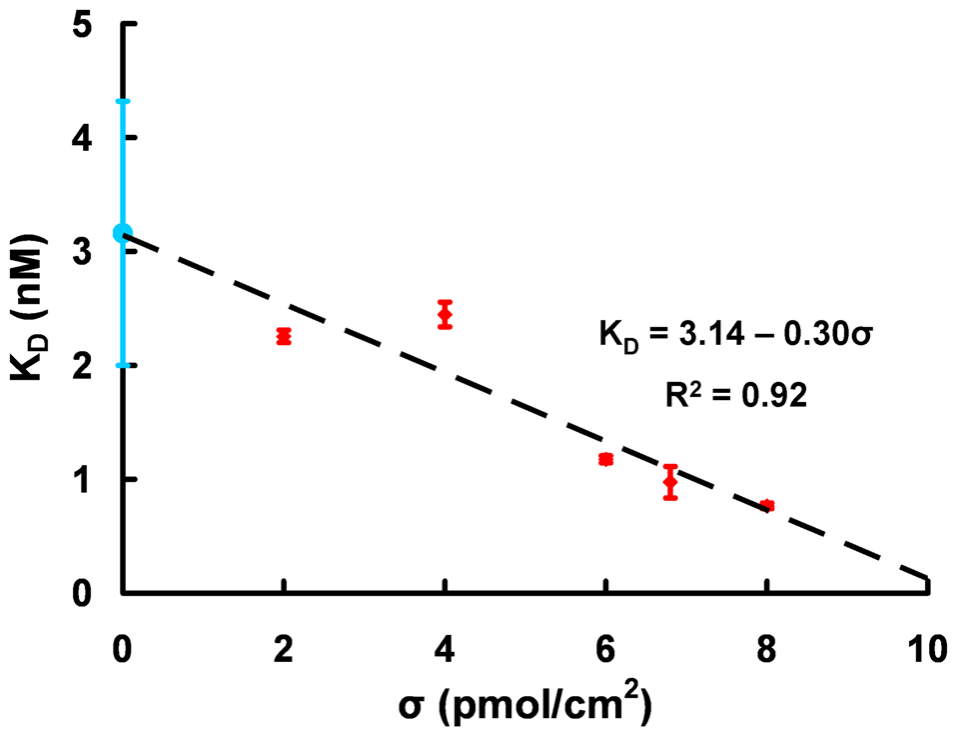
The surface-phase affinity K_D_^Surf^ (in red) extrapolates linearly to the solution-phase affinity K_D_^Sol^ (in blue) at low aptamer grafting density (σ).

Interestingly the surface-phase affinities are significantly different from the solution-phase affinity. As shown in Figure 5, the surface grafting density dependence of K_D_^Surf^ extrapolates linearly at low σ to the solution-phase affinity. This is consistent with a reduction of cooperative interactions between vicinal probes and a lesser impact of the surface compared to the inter-probe or target cooperative interactions. No doubt that the low surface interactions are greatly favored by the use of PEG molecules to form the mixed SAMs leading to very passive surfaces. Furthermore the integration of a methylene and 10-thymine spacer probably ensures that the grafted aptamer maintains a good flexibility to bind thrombin with a similar efficiency as in solution. It should finally be mentioned that both K_D_ values (solution- or surface-phase) were determined assuming a 1∶1 binding stoichiometry. Even if SPR assays alone can not confirm this assumption, the hypothesis is nevertheless reasonable considering that previous studies demonstrated that the aptamer is highly selective towards the heparin-binding exosite of thrombin [31], [36]. In case of multiple affinity sites, like for the Bock's aptamer [33] against thrombin, the kinetic biosensor is not suitable for the quantification of the free protein in competition assay. Therefore this method can not lead to the determination of the dissociation constant in solution in those cases. In fact, a single K_D_ for a multisite protein-ligand interaction would be a misleading definition: as many dissociation constants as active binding sites present on the protein should be defined.

## Conclusion

In conclusion, we have elaborated a label-free kinetic biosensor allowing determination of aptamer-protein affinities both on a surface and in solution. This was possible by taking advantage of quick kinetic interaction on a surface enabling (i) a large linear range of quantification of the free target in solution (from 0.2 to 20 nM), (ii) a low limit of detection (100 pM), and (iii) a short time of detection (less than 10 minutes). The determination of K_D_^Sol^ from competition assay is independent of the interactions occurring at the surface and the corresponding effective surface-phase affinity K_D_^Surf^. This rapid and label-free method is easily extendible to the measurement of various affinities between aptamer and ligands as long as a single binding site of the aptamer towards the ligand is present.
